# Optimization of Biotechnological Vinegar Production from an Algerian Date Variety Using Indigenous Strains and Response Surface Methodology

**DOI:** 10.3390/foods15030518

**Published:** 2026-02-02

**Authors:** Kaouthar Djafri, Toufik Chouana, El Hayfa Khemissat, Meriem Bergouia, Abdelkader Abekhti, Maria D’Elia, Luca Rastrelli

**Affiliations:** 1 Laboratory for the Protection of Ecosystems in Arid and Semi-Arid Zones, Department of Biological Sciences, Faculty of Natural and Life Sciences, Kasdi Merbah University, Ouargla 30000, Algeria; 2 INRAA Experimental Station, National Institute of Agronomic Research of Algeria, Touggourt 30200, Algeria; 3 Laboratory of Natural Saharian Resources, Department of Biological Sciences, Adrar University, Adrar 1001, Algeria; abekhti.aek@univ-adrar.edu.dz; 4 Department of Pharmacy, University of Salerno, 84084 Fisciano, Italy; mdelia@unisa.it; 5 National Biodiversity Future Center—NBFC, 90133 Palermo, Italy; 6 Dipartimento di Scienze della Terra e del Mare, University of Palermo, 90123 Palermo, Italy

**Keywords:** vinegar, Degla Beida date, Central Composite Design (CCD), fermentation, indigenous strains

## Abstract

Vinegar is a traditional fermented food of increasing industrial interest due to its nutritional, sensory, and bioactive properties. This study aimed to develop and optimize a controlled biotechnological process for vinegar production from the Algerian date cultivar Degla Beida, an abundant yet underexploited local resource. Indigenous *Saccharomyces cerevisiae* strains isolated from date fruits and *Acetobacter* sp. strains isolated from traditional date vinegar were employed as starter cultures in a two-stage submerged fermentation process, comprising alcoholic fermentation followed by acetic fermentation. Process optimization was carried out using Response Surface Methodology (RSM) based on a Central Composite Design (CCD), evaluating the effects of initial alcoholic degree (4–10% *v*/*v*) and yeast extract supplementation (0.2–0.5 g/L). The statistical models showed excellent fitting and predictive reliability (*p* < 0.0001; R^2^ = 94.1–99.1%). Under optimal conditions (7% *v*/*v* initial alcohol, 0.2 g/L yeast extract, 30 °C, pH 5), the process yielded a maximum acetic acid concentration of 72 g/L after 11 days, with 80% fermentation efficiency and complete ethanol depletion. The optimized vinegar exhibited enhanced bioactive properties, with a total phenolic content of 620 mg GAE/100 mL and a DPPH radical scavenging activity of 78%, significantly higher than those of the unfermented juice. These results demonstrate the suitability of Degla Beida dates for vinegar production and highlight the potential of indigenous microbial resources for the sustainable valorization of local raw materials through controlled fermentation processes.

## 1. Introduction

Vinegar is a fermented food product of high nutritional and technological value, appreciated worldwide for its distinctive flavor, versatility, and functional properties. Its global market value was estimated at USD 2.27 billion in 2021 and is projected to grow at a compound annual rate of 2.6% by 2027 [1]. Vinegar production involves a two-step bioprocess: the conversion of simple sugars to ethanol by yeasts during alcoholic fermentation, followed by the oxidation of ethanol into acetic acid by acetic acid bacteria (AAB) [2,3]. These microbial reactions enable the bioconversion of complex organic molecules into bioactive and preservable products.

Dates (*Phoenix dactylifera* L.) represent an excellent substrate for vinegar production owing to their high carbohydrate content and abundance of nutrients. Algeria is one of the world’s leading date producers, with an annual production exceeding 1.3 million tons [4], and is characterized by a rich varietal diversity [5]. However, the valorization of this crop remains limited, and large volumes of low-grade dates are underexploited or wasted. Developing biotechnological processes for their transformation into high-value fermented products, such as vinegar, offers a promising avenue for both food security and sustainable waste management.

A broad range of date-based products has been developed, including syrups, pastes, and confectioneries, as well as biotechnological derivatives such as bioethanol and vinegar [6]. Microbial studies on date vinegar have identified multiple acetic acid bacteria species—including *Acetobacter malorum*, *A. persici*, *A. tropicalis*, *Gluconobacter*, *Komagataeibacter xylinus*, *Fructobacillus tropaeoli*, and *Leuconostoc* spp., confirming the microbial diversity and potential of indigenous strains in shaping vinegar quality [7].

Two main fermentation modes are employed for vinegar production: surface and submerged systems. While surface fermentation is traditional and simpler, submerged fermentation provides better control of process parameters, higher microbial activity, and greater acetic acid yields, making it preferable for industrial-scale production [8].

In this context, process optimization, including parameters such as oxygenation, agitation, temperature, and nutrient supply is essential to enhance acetic acid productivity For instance, studies have demonstrated that adjusting the inoculation ratio (e.g., 7:3 *S. cerevisiae* to *A. aceti*) and increasing agitation speed to 160 rpm can significantly boost performance, achieving acetic acid concentrations of up to 6.47%. Furthermore, such optimized conditions have been shown to facilitate the hydrolysis of complex carbohydrates, leading to exceptionally high sugar-to-acetate conversion efficiencies [9].

The use of indigenous starter cultures has emerged as a sustainable and efficient approach to enhance the sensory, nutritional, and safety attributes of fermented foods [10]. Due to their natural adaptation to local substrates and environmental conditions, these microorganisms often exhibit superior fermentation kinetics compared to commercial strains.

For instance, Ref. [11] demonstrated that indigenous *Saccharomyces cerevisiae* strains isolated from Algerian dates outperformed standard reference strains in terms of biomass yield, alcohol production, and fermentation kinetics. In the context of date vinegar production, isolating and characterizing such native *Saccharomyces* and *Acetobacter* strains can ensure process reproducibility and significantly improve biochemical performance under controlled fermentation conditions. Response Surface Methodology (RSM) is a robust statistical and mathematical tool for the optimization of multivariable bioprocesses. It allows for the evaluation of factor interactions and the prediction of optimal conditions with a minimal number of experiments [12]. Response Surface Methodology (RSM) has been extensively implemented to optimize vinegar fermentation across diverse fruit-based substrates, including sugarcane juice [12] and prickly pear [13]. Regarding date-based substrates, Hamden et al. (2022) [2] utilized RSM to fine-tune critical fermentation parameters, demonstrating that the synergistic control of temperature and aeration can significantly maximize acetic acid yields, reaching concentrations of approximately 5.27%. Furthermore, Halladj et al. (2016) [14] employed a Full Factorial Design to evaluate the production of traditional vinegar from Algerian date varieties, highlighting the importance of experimental design in identifying significant process variables. These statistical approaches confirm that RSM and factorial designs are robust tools for predicting interactions between complex factors, thereby substantially enhancing bioconversion efficiency and product consistency compared to traditional spontaneous fermentation methods.

In this context, the present study focuses on the optimization of submerged vinegar fermentation from the Algerian Degla Beida date variety using locally isolated *Saccharomyces cerevisiae* and *Acetobacter* sp. strains as starter cultures. The aim was to identify the optimal conditions for acetic fermentation through Response Surface Methodology coupled with a Central Composite Design (CCD), focusing on two key factors: initial alcoholic degree and nitrogen source enrichment. The study also evaluated the functional properties of the optimized vinegar, including its phenolic composition and antioxidant activity. Overall, this work contributes to the valorization of Algerian date resources through the development of a controlled, reproducible, and high-yielding biotechnological process for vinegar production. To the best of our knowledge, no optimized submerged fermentation using indigenous strains has been reported for Degla Beida vinegar.

## 2. Materials and Methods

### 2.1. Date Fruits and Microbiological Materials

Ripe fruits of the Algerian date cultivar Degla beida (*Phoenix dactylifera* L.) were used as the fermentation substrate. Fruits were harvested at the *Tamar* maturity stage from a date palm oasis located in the Oued-Righ region (Touggourt, southeastern Algeria; 33°06′ N, 6°04′ E). The cultivar is characterized by its firm texture, yellow-brown coloration, and moderate sugar content, making it suitable for traditional vinegar production.

**Figure 1:**
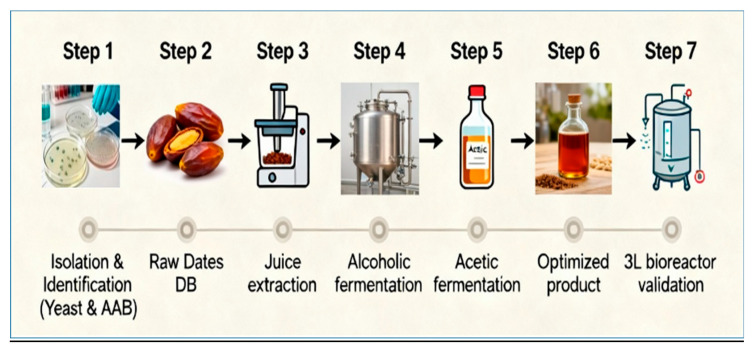
Schematic representation of the experimental workflow for date vinegar. Step 1: Isolation and identification of yeast and acetic acid bacteria (AAB). Step 2: Selection and preparation of ripe Degla Beida dates (DB). Step 3: Extraction of date juice using a soaking–heating process. Step 4: Alcoholic fermentation of date juice with *Saccharomyces cerevisiae*. Step 5: Acetic fermentation with *Acetobacter* sp. VDB7. Step 6: Optimization of fermentation parameters using Response Surface Methodology (RSM). Step 7: Validation of the optimized process.

After harvesting, the fruits were immediately transported to the laboratory under refrigerated conditions (4 ± 1 °C) and processed within 24 h (Figure 1).

### 2.2. Chemicals and Culture Media

All chemicals and analytical-grade reagents were purchased from Sigma-Aldrich (St. Louis, MO, USA), PanReac AppliChem (Barcelona, Spain), and Merck KGaA (Darmstadt, Germany), unless otherwise stated. Distilled and deionized water (resistivity ≥ 18.2 MΩ·cm, Milli-Q, Millipore, Burlington, MA, USA) was used for all analytical and culture media preparations. Analytical solvents used for phenolic extraction and spectrophotometric analyses included methanol (≥99.9%, HPLC grade, Sigma-Aldrich, Saint Louis, MO, USA), ethanol (≥99.8%, analytical grade, Merck, Darmstadt, Germany), acetone (≥99.5%, PanReac, Barcelona, Spain), and sulfuric acid (H_2_SO_4_, 95–97%, Merck). Sodium hydroxide (NaOH, ≥98%), phenolphthalein indicator, gallic acid standard (≥98%), Folin–Ciocalteu reagent, and 2,2-diphenyl-1-picrylhydrazyl (DPPH, ≥95%) were supplied by Sigma-Aldrich. All other chemicals and mineral salts were of analytical purity and used without further purification. Culture media for microbial isolation and propagation were prepared as follows: Carlsberg medium for yeast cultivation, composed of yeast extract (3.0 g/L), peptone (5.0 g/L), and glucose (20.0 g/L); Carr medium for Acetobacter enrichment, containing yeast extract (3.0 g/L), peptone (1.0 g/L), and glucose (30.0 g/L); GYC medium, used for selective isolation of acetic acid bacteria, composed of glucose (100 g/L), yeast extract (10 g/L), and calcium carbonate (20 g/L) as neutralizing agent. All media components were obtained from Biokar Diagnostics (Beauvais, France) and Oxoid Ltd. (Hampshire, UK). Media were sterilized by autoclaving at 121 °C for 15 min before inoculation.

### 2.3. Extraction and Preparation of Date Juice for Fermentation

Date juice was prepared using a soaking-based extraction method adapted from [15,16], with minor modifications to improve extraction yield and compositional quality. Briefly, 1.0 kg of washed, pitted, and ground DB dates was mixed with 2.5 L of distilled water and heated in a thermostatic water bath at 70 °C for 40 min under continuous mechanical stirring. After cooling to room temperature, the mixture was filtered through a fine cotton cloth and centrifuged at 4000 rpm for 10 min to remove suspended solids. The supernatant was pasteurized at 75 °C for 30 s to reduce microbial load while preserving the nutritional integrity of the juice. The clarified juice was used fresh for fermentation trials without further storage.

Physicochemical analyses included the determination of pH, °Brix, titratable acidity, total and reducing sugars, protein content, total soluble solids (TSS), total polyphenols, and mineral composition, as described in Section 2.6.

### 2.4. Yeast Isolation and Identification

*Saccharomyces* yeasts were isolated from ripe DB Degla beida date fruits and used as starter cultures for alcoholic fermentation trials. Genomic DNA was extracted from yeast cultures following cell lysis, phenol–chloroform deproteinization, and isopropanol precipitation according to [17].

Yeast identification was performed by real-time PCR (qPCR). The PCR reaction mixture (25 µL final volume) contained SuperMix and purified genomic DNA as template. Two primer pairs were used: the universal primers ITS1F (5′-GCTGCAACCATGGACTGGAT-3′) and ITS1R (5′-TCRATGGTGAAGTCAACGTG-3′), and the *Saccharomyces cerevisiae*-specific primers SCF1 (5′-ATACCCTTCTTAACACCTGGC-3′) and SCR1 (5′-GGACTCTGGACATGCAAGAT-3′). Thermal cycling conditions were set according to [18] and consisted of enzymatic activation followed by 40 amplification cycles, including denaturation, annealing, and elongation steps.

### 2.5. Acetic Acid Bacteria Isolation and Selection

Acetic acid bacteria (AAB) were isolated from a traditionally prepared vinegar obtained from the Degla Beida date cultivar. The inoculum source consisted of the “mother of vinegar”, a surface biofilm naturally formed during spontaneous acetic fermentation. Primary isolation was carried out on Carr and GYC media, and colonies exhibiting typical AAB features were selected and purified through repeated sub-culturing.

The isolates were subjected to phenotypic screening, including observation of colony morphology and assessment of growth behavior and acetic acid production during small-scale fermentation assays. Based on fermentation performance and comparison with literature descriptions, five promising bacterial strains were selected and tentatively assigned to the genera *Gluconobacter* and *Acetobacter* [19,20]. These strains were designated VDB1, VDB5, VDB7, VDB60, and VDB45.

Among them, strain VDB7 (*Acetobacter* sp.) exhibited the highest acetic acid production and growth performance and was therefore selected for subsequent optimization and scale-up studies. Molecular identification of acetic acid bacteria was beyond the scope of the present study and will be addressed in future work; strain selection was therefore based on phenotypic traits and fermentation performance.

### 2.6. Optimization of the Fermentation Process

A sequential optimization strategy was applied, starting with the screening of factors affecting both alcoholic and acetic fermentations, followed by process optimization using Response Surface Methodology (RSM) with a Central Composite Design (CCD). Preliminary screening comprised 52 experimental trials for alcoholic fermentation, testing six independent variables: initial °Brix, temperature (°C), fermentation time (h), inoculum size (% *v*/*v*), and nitrogen supplementation (mineral and organic sources). The most significant parameters influencing ethanol yield were initial °Brix and yeast-extract concentration. A subsequent screening for acetic fermentation evaluated temperature, fermentation time, initial alcohol content (% *v*/*v*), inoculum size (% *v*/*v*), yeast-extract level (g L^−1^), and oxygenation. Alcohol concentration and yeast extract were retained as the two critical variables for further optimization.

#### 2.6.1. Alcoholic Fermentation

The yeast inoculum was prepared in Carlsberg medium and incubated at 28 °C with agitation (45 rpm) until an optical density (OD_600_) of 0.6 was reached. Fermentations were conducted under semi-anaerobic conditions in 500 mL Erlenmeyer flasks containing 200 mL of date juice adjusted to 15–25 °Brix and supplemented with 0.3–0.5 g L^−1^ yeast extract. The flasks were inoculated with 10% (*v*/*v*) of the activated *S. cerevisiae* culture and maintained at pH 5.0. Ethanol production was monitored daily by refractometry and spectrophotometry as described in Section 2.6.2.

#### 2.6.2. Acetic Fermentation and Response Surface Design

Optimization of the acetic-acid fermentation step was carried out using a Central Composite Design (CCD) with two independent variables: initial ethanol concentration (X_1_, 4–10% *v*/*v*) and yeast-extract concentration (X_2_, 0.2–0.5 g L^−1^). Thirteen experimental runs were generated, including five center points to estimate pure error. Fermentations were performed at 30 °C and pH 5.0 under continuous agitation (45 rpm) for up to 16 days. Acetic-acid content and residual ethanol were measured at 4-day intervals.

Experimental data were fitted to a second-order polynomial model (Equation (1)):

*Y* = β_0_ + ∑β_i_X_i_ + ∑β_ii_X_i_^2^ + ∑β_ij_X_i_X_j_ (1)

where *Y* is the predicted response and *X*_1_ and *X*_2_ are the coded independent variables.

Statistical analyses and model validation were performed using Minitab 18 (Minitab Inc., State College, PA, USA). Model adequacy was assessed through analysis of variance (ANOVA), coefficient of determination (R^2^ and adjusted R^2^), *p*-values (<0.05), and lack-of-fit tests.

#### 2.6.3. Acetic Fermentation Procedure

The *Acetobacter* sp. strain (VDB7) was reactivated in Carr broth composed of 3.0% (*w*/*v*) yeast extract, 1.0% (*w*/*v*) peptone, and 3.0% (*w*/*v*) glucose. For inoculum preparation, sterile 100 mL Erlenmeyer flasks containing 50 mL of Carr broth were inoculated with the reactivated culture and incubated at 30 °C under orbital agitation (150 rpm) until an optical density (OD_600_) of 0.6 was reached. Cell density was estimated based on OD_600_ measurements, where an OD_600_ value of 0.6 corresponded approximately to 4.8 × 10^8^ CFU mL^−1^ for *Acetobacter* sp. and between 5 × 10^6^ and 5 × 10^7^ CFU mL^−1^ for yeast cells, assuming proportionality between optical density and viable cell count [21].

The alcoholic date broth obtained from the previous fermentation step was adjusted to an ethanol content of 5–10% (*v*/*v*) according to the optimized conditions. Fermentations were conducted in 500 mL Erlenmeyer flasks containing 200 mL of medium, inoculated with 5% (*v*/*v*) of the prepared *Acetobacter* sp. culture. The process was carried out at 30 °C and pH 5.0 under continuous agitation (45 rpm) using a Variomag magnetic stirrer (Thermo Fisher Scientific, Bremen, Germany).

Total titratable acidity and residual ethanol were measured every 4 days to monitor the progression of acetic acid fermentation and evaluate conversion efficiency.

### 2.7. Date Juice Characterization

Standard AOAC (1990) methods were used to determine pH, total acidity, moisture, and ash content [22]. Total and reducing sugars were quantified using the Bertrand method, after a defecation step, where 100 mL of the date must was treated with 10% lead acetate and neutralized with 1 g sodium carbonate (Na_2_CO_3_). For reducing sugars, the clarified extract was reacted with an excess of alkaline copper solution and boiled for 3 min. The resulting cuprous oxide (Cu_2_O) precipitate was separated, dissolved in an acidic ferric sulfate solution, and titrated against 0.1 N potassium permanganate (KMnO_4_). For total sugars, an acid hydrolysis step was performed by adding 5 mL of HCl to 50 mL of the clarified juice at 70 °C for 12 min to convert non-reducing sugars into reducing units, followed by the same titration procedure [23].

The concentrations of individual sugars were quantified using a Shimadzu HPLC system equipped with a Refractive Index Detector (RID-20A) and a Shim-pack GIST NH2 column (250 × 4.6 mm, 5 µm). The analysis followed the protocol described by El-Sohaimy et al. [24] with some modifications. The chromatographic conditions were as follows: the mobile phase consisted of an Acetonitrile/Ultra-pure Water mixture (80/20, *v*/*v*), the pump flow rate was set at 1.0 mL/min, and the column temperature was maintained at 35 °C using a CTO-20A oven. Prior to analysis, samples were filtered through a 0.22 µm syringe filter, and an injection volume of 10 µL was used. Protein content was determined by the Kjeldahl method [22].

The total phenolic content was estimated following the Al Juhaimi et al. [25] protocol, with minor modifications, using the Folin–Ciocalteu method. Briefly, 1 mL of sample was vigorously mixed with 1 mL of Folin–Ciocalteu reagent (diluted 10-fold) for 5 min. Subsequently, 10 mL of sodium carbonate solution (200 g/L) was added, and the mixture was adjusted to a final volume of 25 mL. The reaction mixture was incubated for 2 h in the dark at room temperature. The absorbance was measured at 765 nm using a Cary 100 UV-Vis spectrophotometer (Agilent Technologies, Santa Clara, CA, USA). Results were expressed as milligrams of gallic acid equivalents per 100 g of dry matter (mg GAE/100 g DM).

The minerals were determined according to the method described by AOAC [22] with slight modifications. The contents of Na, K, Ca, Mg, Fe, Zn, Cu, and Mn were determined using High-Resolution Continuum Source Flame Atomic Absorption Spectrometry (HR-CS FAAS) with an Analytik Jena contrAA 800D spectrometer (Jena, Germany). A volume of 10 mL of date juice was first reduced to ash, which then underwent wet mineralization using a mixture of HNO_3_/H_2_SO_4_ at 180 °C until a clear solution was obtained. The digested sample was subsequently diluted to a final volume of 50 mL. Measurements were performed at the specific resonance wavelengths for each element, and concentrations were quantified using standard calibration curves (R^2^ ≥ 0.99).

### 2.8. Fermentation Analyses

Titratable acidity was determined by titration with 0.1 N NaOH to pH 8.2, expressed as g acetic acid/L [1]. °Brix values were measured using a digital refractometer (Bellingham + Stanley, Tunbridge Wells, UK). Ethanol content was determined spectrophotometrically after micro-distillation, using dichromate oxidation [26,27]. Calibration was linear from 0–15% *v*/*v* (R^2^ = 0.992). Fermentation efficiency (FE) and acetic acid productivity (AAP) were calculated according to [8,28], Equations (2) and (3):

FE(%) = [Acetic acid]a_ctual_/[Acetic acid]_theoretical_ × 100 (2)

AAP = [Acetic acid]_final_/Fermentation time (3)

### 2.9. Polyphenols and Antioxidant Activity

Total phenolic content of optimized vinegar was measured as described above. The scavenging activity of free radicals in the vinegar samples was evaluated using the 1,1-diphenyl-2-picrylhydrazyl (DPPH) radical [29,30]. A methanolic solution of DPPH (0.1 mM) was prepared by dissolving 4 mg of DPPH in 100 mL of methanol. To 1 mL of each vinegar sample, 1 mL of the DPPH solution was added. The mixture was then incubated in the dark for 30 min at room temperature. The absorbance was measured at 517 nm using a spectrophotometer. The antioxidant activity, related to the scavenging effect of the DPPH radical, is expressed as the percentage of inhibition (PI), calculated using the following formula:

%PI = (A control − A test/A control) × 100

### 2.10. Statistical Analysis

All experiments were carried out in triplicate, and the results are presented as mean ± standard deviation (SD). Statistical analyses were performed using Minitab 18 (Minitab Inc., State College, PA, USA). The effects of the independent variables on the responses were evaluated by analysis of variance (ANOVA) at a significance level of *p* < 0.05. Model adequacy and goodness of fit were assessed based on the coefficient of determination (R^2^), adjusted R^2^, and lack-of-fit test. Response Surface Methodology (RSM) was employed to determine the optimal conditions for acetic fermentation using a Central Composite Design (CCD). Regression coefficients were estimated through the least-squares method, and three-dimensional surface and contour plots were generated to visualize factor interactions and identify the experimental region corresponding to the maximum acetic-acid yield.

## 3. Results and Discussion

### 3.1. Physicochemical Characteristics of Date Juice as a Fermentation Medium

The efficiency of any fermentation process is strongly influenced by the quality and composition of the culture medium [31]. For this reason, a detailed physicochemical characterization of Degla beida date juice was carried out to evaluate its suitability as a substrate for sequential alcoholic and acetic fermentations. The results are summarized in Table 1.

The date juice was characterized by a high total sugar content (25.61 ± 0.03%), consisting mainly of sucrose (12.80 ± 0.22%) and reducing sugars (10.13 ± 0.12%). Minor amounts of xylose and galactose were also detected, providing readily assimilable carbon sources for fermentative microorganisms. This sugar profile confirms the high fermentability of the substrate and supports its suitability for ethanol production, as previously reported for date-based fermentation media.

The juice exhibited a moderately acidic pH (5.31 ± 0.01) and a titratable acidity of 0.95 ± 0.02%, conditions that are favorable for yeast activity during alcoholic fermentation and do not inhibit the subsequent development of acetic acid bacteria. Conversely, the protein content was relatively low (0.24 ± 0.03%), indicating limited nitrogen availability. This level is insufficient to fully support microbial growth and metabolic activity, thus justifying the supplementation of an external nitrogen source to ensure optimal fermentation performance [32].

Regarding mineral composition, the juice contained appreciable amounts of calcium (310 mg/100 mL), potassium (280 mg/100 mL), and sodium (295 mg/100 mL), together with magnesium (55 mg/100 mL), iron (2.10 mg/100 mL), zinc (0.28 mg/100 mL), copper (0.05 mg/100 mL), and manganese (0.07 mg/100 mL). These minerals are known to play a crucial role as enzymatic cofactors and regulators of cellular metabolism, contributing to microbial growth and biochemical reactions during acetic fermentation [33,34].

The total phenolic content of the date juice reached 572 mg GAE/100 g of dry matter, a value higher than those previously reported in [35] for the same cultivar, while remaining comparable to those observed in other Algerian date varieties such as ‘Tinissine’ and ‘Kentichi’. Similar values have also been reported [36] for Degla Beida. The high phenolic content confers significant antioxidant and functional properties to the juice and, consequently, to the resulting vinegar. This finding is consistent with previous studies highlighting the strong bioactive potential of DB dates [37,38].

Overall, the favorable sugar profile, adequate acidity, rich mineral composition, and high phenolic content make Degla Beida date juice a highly suitable substrate for microbial growth, enzymatic activity, and the efficient production of ethanol and acetic acid. These characteristics confirm its strong potential for biotechnological valorization in vinegar production, in agreement with previous reports on date-based fermentation processes [39,40].

### 3.2. Molecular Identification PCR Analysis

**Figure 2:**
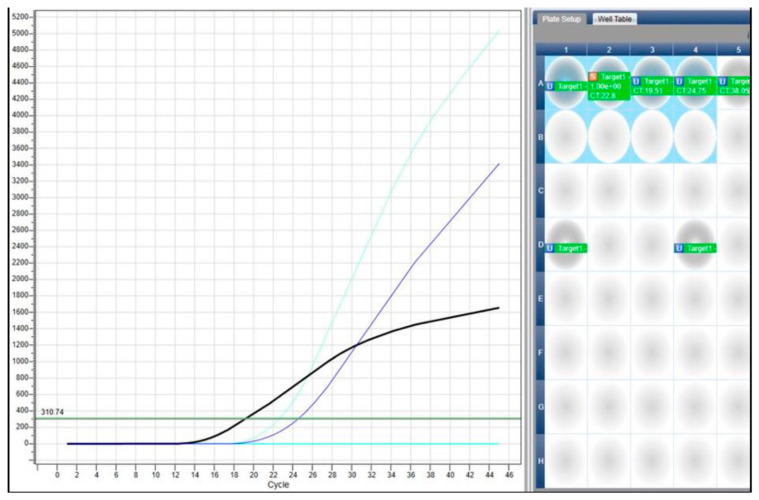
Representative qPCR amplification curve with Ct determination of *Saccharomyces cerevisiae*.

The melting curves resulting from real-time PCR amplification displayed a single peak for each isolate, with melting temperatures (Tm) closely matching those of the *S. cerevisiae* positive control strain (Figure 2). The obtained curve profiles were typically sigmoidal, with cycle threshold (Ct) values within the acceptable range for the extracted DNA, revealing successful amplification, template integrity, and absence of PCR inhibition. Specifically, Figure 2 highlights a significant reduction in the cycle threshold (Ct = 19.51) compared to the control (Ct = 22.80), indicating sufficient biomass concentration. Slight variability in Ct values observed among isolates is commonly reported in real-time PCR assays and may result from differences in DNA isolation efficiency as well as the physiological state of the cells during extraction. Similar results were obtained with both primer pairs, namely the ITS1F/ITS1R universal set and the SCF1/SCR2 S. cerevisiae-specific primers. Hence, the concordant species-specific amplification, single-peak melting profiles, and ITS sequence homology confirmed that the selected starter candidates belonged to *Saccharomyces cerevisiae* and demonstrated their high metabolic potential prior to use as a starter culture for vinegar production from date must.

### 3.3. Optimization of Acetic Fermentation by Central Composite Design (CCD)

#### 3.3.1. CCD Experimental Design and Model Adequacy

The Central Composite Design (CCD) was applied to evaluate the combined effects of initial alcoholic degree (X_1_) and yeast extract concentration (X_2_) on acetic acid production and residual ethanol. The experimental design and measured responses are reported in Table 2.

#### 3.3.2. Response Surface Model Fitting and Statistical Validation

The suitability of the quadratic regression models was confirmed by high F-values (25–157) and very low *p*-values (*p* < 0.0001). Coefficients of determination (R^2^) ranged from 91.35% to 99.66%, with adjusted R^2^ values between 85.17% and 99.43%, indicating a strong correlation between process variables and responses. The low coefficients of variation further confirmed the high precision and reproducibility of the experimental data (Table 3).

#### 3.3.3. Effect of Initial Alcoholic Degree and Yeast Extract on Acetic Acid Production

**Figure 3:**
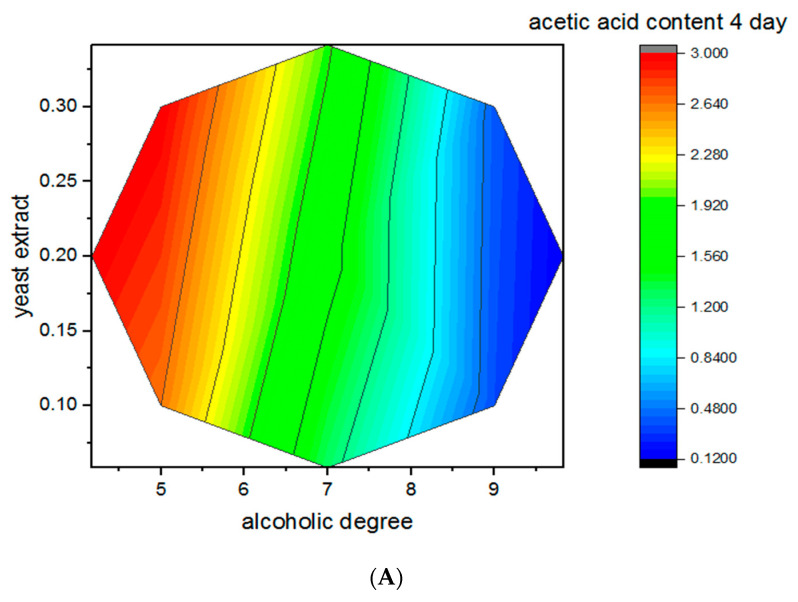

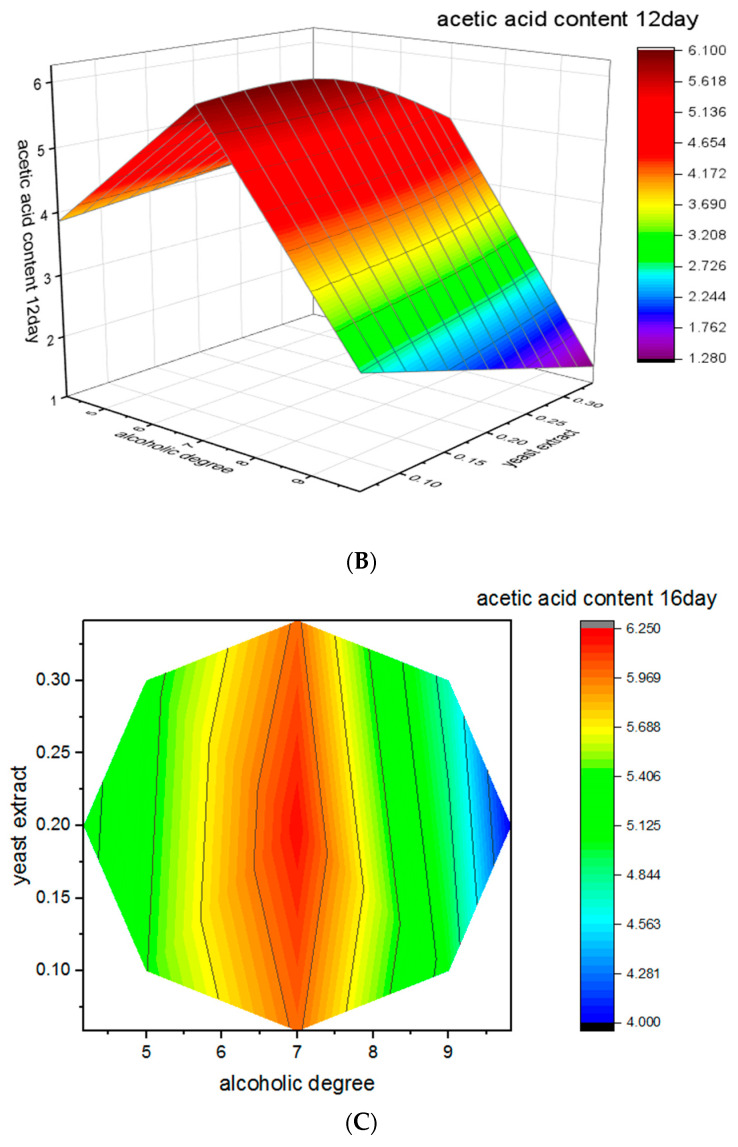
Three-dimensional response surface plots illustrating the combined effect of initial alcoholic degree (% *v*/*v*) and yeast extract concentration (g L^−1^) on acetic acid content (% *w*/*v*) during acetic fermentation of Degla Beida date juice at different fermentation times: (**A**) 4 days, (**B**) 12 days, and (**C**) 16 days.

The interaction between initial alcoholic degree and yeast extract concentration is illustrated by the three-dimensional response surfaces shown in Figure 3A–C.

The surfaces were generated from the second-order polynomial models obtained.

During the first 4 days of fermentation, acetic acid production ranged from 0.12 to 3.00% (*w*/*v*), showing predominantly linear effects. This linear trend suggests that the acetic acid bacteria were in an early adaptation phase, experiencing minimal metabolic stress as the fermentation proceeded in a predictable and gradual manner. From day 8 onward, acetic acid content increased markedly, reaching values above 5.0% (*w*/*v*) under optimal conditions. Maximum acidity (>6.0%) was achieved after 8 days at an initial alcoholic degree between 6–7% and yeast extract concentrations around 0.20–0.30 g/L. At higher initial alcohol levels (9–9.82% *v*/*v*), acetic acid production was significantly reduced, confirming ethanol inhibition effects on acetic acid bacteria. The quadratic effect of initial ethanol, illustrated by the curvature in the response surfaces, reflects the biological threshold of the indigenous strains. While ethanol serves as the primary substrate, its excess triggers osmotic stress and destabilizes the bacterial cell membrane, explaining the decline in productivity beyond the optimal threshold. These results were consistent with previous findings [41], which reported optimal acidity values around 4.8% in onion vinegar at moderate ethanol concentrations, and aligned with observations in nipa sap vinegar [42]. The positive effect of yeast extract was evident in runs 3 and 13, where increased nitrogen availability enhanced acetic acid production. Yeast extract provided assimilable nitrogen, vitamins, and growth factors, improving microbial viability and metabolic activity, as previously established in the research [43].

#### 3.3.4. Effect of Process Variables on Residual Ethanol Consumption

**Figure 4:**
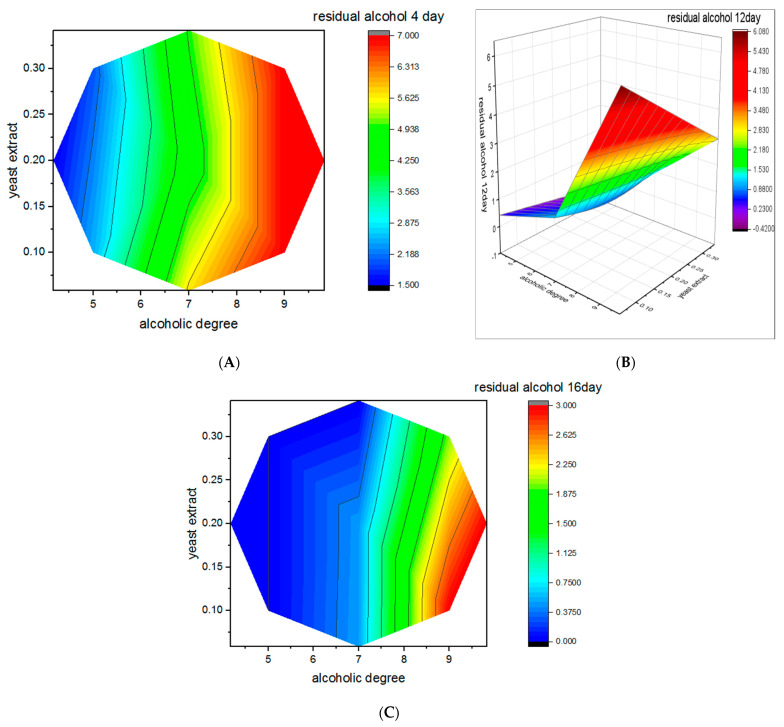
Three-dimensional response surface plots showing the combined effect of initial alcoholic degree (% *v*/*v*) and yeast extract concentration (g L^−1^) on residual alcohol content (% *v*/*v*) during acetic fermentation of Degla Beida date juice at different fermentation times: (**A**) 4 days, (**B**) 12 days, and (**C**) 16 days. The response surfaces were generated using second-order polynomial models derived from Response Surface Methodology (RSM). The plots illustrate the progressive decrease in residual alcohol as fermentation proceeds, with a marked reduction at moderate initial alcoholic degrees (approximately 6–7% *v*/*v*) and adequate yeast extract supplementation, confirming efficient ethanol oxidation by acetic acid bacteria under optimized conditions.

Residual ethanol levels varied significantly depending on initial alcoholic degree and yeast extract concentration (Figure 4).

After 4 days of fermentation, residual ethanol ranged between 1.5% and 7.0% (*v*/*v*), declining significantly to 0–0.5% by day 16. Complete ethanol depletion (0% *v*/*v*), reflecting maximum accumulation of acetic acid, was specifically achieved in runs 2, 3, 9, 10, and 13. This phenomenon can be explained by the interplay of multiple factors affecting microbial metabolic activity and substrate bioconversion. Similar studies have demonstrated that employing these specific strains in date vinegar fermentation facilitates highly efficient bioconversion, ensuring a final product that meets international quality standards within a record timeframe. In this regard, the high fermentation efficiency observed in our study is consistent with the work of Al-Kharousi et al. [44] regarding the use of *Acetobacter* strains for date vinegar production, as well as the findings of Nosratabadi et al. [7] involving the application of *Komagataeibacter xylinus* in the same substrate, while most other trials showed residual values below 0.5% *v*/*v*, complying with Codex Alimentarius standards. Moderate initial alcohol levels (6–7% *v*/*v*) allowed almost complete ethanol conversion, whereas high concentrations (>9%) resulted in residual ethanol > 3 g/100 mL even after 16 days, indicating metabolic inhibition. Conversely, low initial alcohol levels (4–5%) led to rapid ethanol depletion but limited final acidity (<5°).

**Figure 5:**
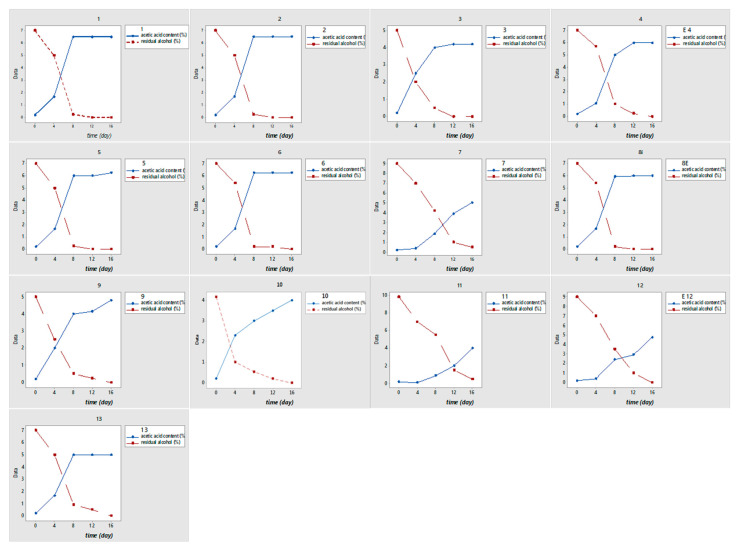
Line plots showing the evolution of acetic acid concentration (% *w*/*v*) and residual alcohol content (% *v*/*v*) during acetic fermentation of Degla Beida date wine under different experimental conditions of initial alcoholic degree and yeast extract supplementation, as defined by the Central Composite Design. Each panel corresponds to an individual experimental run (Runs 1–13) and reports the kinetic profiles monitored at 4, 8, 12, and 16 days of fermentation. The plots highlight the progressive conversion of ethanol into acetic acid, with a rapid decrease in residual alcohol occurring mainly during the first 8 days, followed by a stabilization phase. Differences among runs reflect the combined influence of initial ethanol concentration and nitrogen availability on fermentation kinetics and metabolic efficiency of *Acetobacter* sp.

These findings align with [44], who reported negligible residual ethanol in optimized date vinegars. The observed kinetic profiles (Figure 5) clearly demonstrate the efficiency of the optimized conditions.

#### 3.3.5. Fermentation Efficiency and Acetic Acid Productivity

Fermentation efficiency (EF) and acetic acid productivity (AAP) calculated at different fermentation times (4, 8, 12, and 16 days) under the experimental conditions defined by the Central Composite Design are summarized in Table 4. These parameters provide complementary information on the effectiveness of ethanol conversion into acetic acid and on the kinetics of the process, respectively.

Overall, EF values showed a marked dependence on both the initial alcoholic degree and yeast extract concentration, as well as on fermentation time. At moderate initial ethanol levels (5–7% *v*/*v*) combined with yeast extract supplementation around 0.20 g L^−1^, EF increased rapidly during the first 8 days of fermentation, reaching values between 64.8 and 71.6% (runs 1, 2, 5, 6, and 8). For these conditions, EF tended to stabilize or slightly decrease thereafter, remaining in the range of 63–69% at day 16, indicating that most of the ethanol had already been efficiently converted by day 8–12. The rapid increase in EF during the first 8 days can be attributed to the optimization of fermentation parameters, which promoted exponential bacterial growth and high enzymatic efficiency of *Acetobacter* at the early stages. This stabilization observed thereafter is likely due to the depletion of the ethanol substrate. These findings align with Al-Kharousi et al. [44], who demonstrated that optimized conditions and starter culture-based production can yield acetic acid levels meeting international standards (4.67%) while dramatically reducing fermentation time from 40 days to 4 days.

Among the tested conditions, runs 2 and 6 (7.00% *v*/*v* initial alcohol and 0.20 g L^−1^ yeast extract) exhibited the highest and most consistent performance. In particular, run 2 reached an EF of 71.63% at day 8, which remained close to 69% at day 12 and day 16, confirming the robustness of these operating conditions. Similar trends were observed for run 6, with EF values of 70.99% (day 4) and 68.28% (day 8–12). These results clearly indicate that a balanced combination of ethanol availability and assimilable nitrogen favors optimal acetic metabolism.

In contrast, extreme initial alcoholic degrees negatively affected fermentation efficiency. At high ethanol concentrations (9.00–9.82% *v*/*v*; runs 7, 11, and 12), EF values were very low at the early stages (≤12.4% at day 8 for run 11) and increased only gradually over time, reaching at most 43.6% (run 7) and 38.7% (run 12) after 16 days. This behavior suggests partial inhibition or delayed adaptation of acetic acid bacteria at elevated ethanol levels. Conversely, at low initial alcohol content (4.17% *v*/*v*; run 10), EF values increased steadily over time, reaching 69.94% at day 16, but remained lower during the early stages, reflecting limited substrate availability for rapid acetic acid formation. This behavior suggests partial inhibition or delayed adaptation of acetic acid bacteria at elevated ethanol levels. Conversely, at low initial alcohol content (4.17% *v*/*v*; run 10), EF values increased steadily over time, reaching 69.94% at day 16, but remained lower during the early stages, reflecting limited substrate availability for rapid acetic acid formation [20]. These trends can be explained by the dual effect of ethanol on Acetobacter metabolism; while high concentrations (>9%) act as a stress factor that disrupts the bacterial cell membrane and inhibits the Alcohol Dehydrogenase (ADH) enzyme system [45], the lower concentration in run 10 fails to reach the enzyme’s saturation point, thereby limiting the initial bioconversion rate.

Trends observed for acetic acid productivity (AAP) were consistent with those of EF but provided additional insight into process kinetics. The highest AAP values were generally recorded at day 8, corresponding to the exponential phase of acetic acid production. Maximum productivities of 7.85–7.88 g L^−1^ day^−1^ were achieved in runs 1 and 2 (7.00% *v*/*v* ethanol, 0.20 g L^−1^ yeast extract), while run 6 reached 7.56 g L^−1^ day^−1^ at the same time point. After day 8, AAP progressively decreased in all experiments, dropping to values below 4 g L^−1^ day^−1^ at day 16, which is indicative of substrate depletion and reduced metabolic activity.

At high ethanol levels, AAP values were markedly lower during the initial stages (≤0.88 g L^−1^ day^−1^ at day 8 for run 11), increasing only at later stages as the microbial population adapted to the stressful conditions. Conversely, low ethanol levels (run 10) led to relatively high AAP at day 4 (5.25 g L^−1^ day^−1^), followed by a rapid decline, confirming that insufficient ethanol limits sustained acetic acid production. Taken together, the results reported in Table 4 clearly demonstrate that an initial alcoholic degree of approximately 7% (*v*/*v*) combined with yeast extract supplementation around 0.20 g L^−1^ represents the optimal compromise between high fermentation efficiency and elevated acetic acid productivity. These conditions ensure rapid ethanol conversion, high acid yield, and stable performance over time. The observed behavior is in agreement with previous studies reporting that moderate ethanol concentrations and adequate nitrogen availability are critical for maximizing acetic fermentation efficiency and productivity in fruit-based vinegars. Finally, these findings also emphasize the importance of cultivar selection, as the Degla Beida date juice provided a favorable compositional matrix that supported efficient microbial activity and high conversion yields, in line with previous reports highlighting the strong biotechnological potential of this cultivar for vinegar production [3].

### 3.4. Validation and Functional Properties of Optimized Date Vinegar

Validation experiments performed in a 3 L bioreactor confirmed the robustness and scalability of the optimized fermentation conditions identified by Response Surface Methodology. Under controlled operating conditions, the acetic acid concentration increased from 72.0 to 74.2 g L^−1^, demonstrating the effectiveness of optimized aeration. Consistent with the findings of [14], rigorous control of the fermenter design, specifically the lid perforation diameter and the headspace-to-interface ratio, promotes optimal oxygen mass transfer to the liquid core while eliminating anoxic zones. This adapted air volume acts as a physical catalyst, enabling the *Acetobacter* strain to reach its full metabolic potential without respiratory limitations, thereby ensuring maximum bioconversion, while ethanol was completely consumed, demonstrating effective process control and high conversion efficiency at a larger scale. These results are in good agreement with those obtained at flask level, confirming the reliability of the optimization strategy.

**Figure 6:**
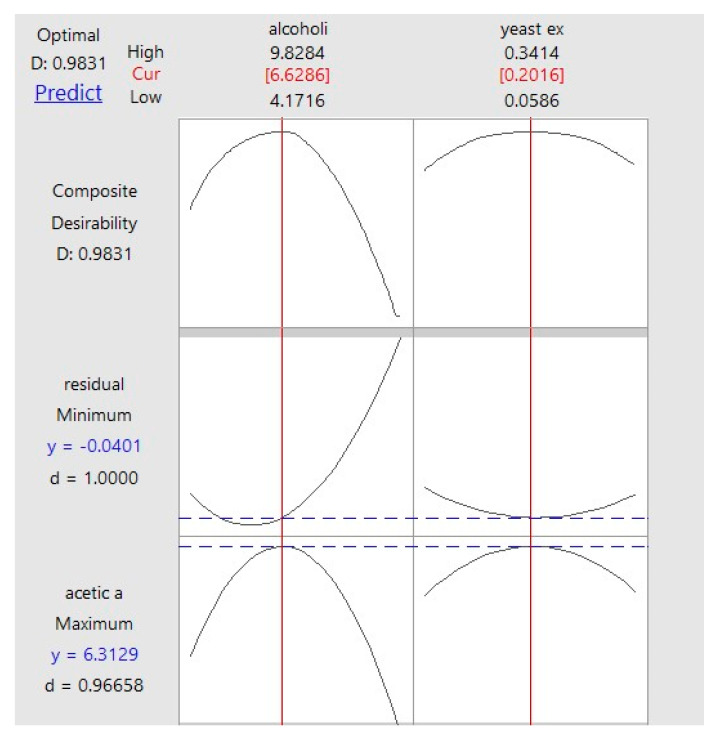
Desirability and prediction curves from the response optimizer.

The desirability function analysis further supported these findings, indicating an overall desirability value close to unity, corresponding to an optimal compromise between maximum acetic acid production and minimal residual alcohol (Figure 6).

Beyond process performance, acetic fermentation led to a clear enhancement of the functional properties of the final product (Table 5). A significant increase in total phenolic content was observed, rising from 57.5 ± 0.34 mg GAE/100 mL in date juice to 62.0 ± 0.09 mg GAE/100 mL in the optimized date vinegar. This enrichment was accompanied by a marked improvement in antioxidant activity, which increased from 69.4% to 78.0% as determined by the DPPH radical scavenging assay.

The parallel increase in phenolic content and antioxidant capacity indicates that acetic fermentation promoted the release and/or transformation of phenolic compounds into more bioactive forms. Such behavior is commonly attributed to enzymatic hydrolysis and oxidative reactions occurring during fermentation, which can liberate bound phenolics from complex matrices and enhance their antioxidant effectiveness. The statistically significant correlation between total phenolics and antioxidant activity (*p* < 0.05) confirms that polyphenols are the main contributors to the observed functional enhancement.

These findings are consistent with previous reports showing a strong relationship between phenolic content and antioxidant activity in date-derived products, as well as in other fermented beverages. The research of Velioglu [46] demonstrated a direct correlation between antioxidant capacity and total phenolics, flavonoids, and carotenoids, while similar improvements during fermentation have been reported for soursop juice transformed into wine [47]. Overall, the combined results highlight that the optimized acetic fermentation process not only ensures high acetic acid yield and complete ethanol depletion but also enhances the nutritional and functional quality of Degla Beida date vinegar, reinforcing its potential as a value-added fermented product.

## 4. Conclusions

This study successfully demonstrates the biotechnological valorization of the Algerian Degla Beida date cultivar as a high-potential substrate for vinegar production. By integrating the selection of indigenous microorganisms (*Saccharomyces cerevisiae* and *Acetobacter* sp.) with Response Surface Methodology (RSM), we developed a robust fermentation process that maximizes acetic acid yield and nutritional quality.

This study aims to highlight the efficiency of utilizing locally adapted microbial strains, which ensured an efficient conversion of the specific sugar profile characteristic of Degla Beida dates for vinegar production. The optimized process not only achieved high fermentation kinetics but also significantly enhanced the functional profile of the final product, notably increasing the total phenolic content and antioxidant capacity compared to the raw juice. Furthermore, a significant achievement of this work is the successful validation of the fermentation process through the transition from Erlenmeyer flasks to a laboratory-scale bioreactor. This progression represents a crucial step forward, as it demonstrates the stability and efficiency of the indigenous strains under controlled parameters.

Despite these promising results, it is important to acknowledge certain limitations. While this study provided a thorough phenotypic characterization and performance evaluation of the indigenous acetic acid bacteria, further genotypic identification is required to confirm the species-level taxonomy. Additionally, future research should incorporate a professional sensory evaluation (organoleptic analysis) conducted by a panel of experts to assess the flavor profile, aroma, and consumer acceptability of the produced date vinegar. 

Overall, this work demonstrates a sustainable approach to transforming agricultural resources into high-value functional foods, laying the groundwork for a specialized fermentation sector.

## Figures and Tables

**Table 1:** Physicochemical and mineral composition of Degla Beida date juice.

Parameter	Unit	Value
Water content	% (FW)	76.00 ± 0.02
pH	–	5.31 ± 0.10
Titratable acidity	% (FW) *	0.95 ± 0.02
Reducing sugars	% (FW)	10.13 ± 0.12
Sucrose	% (FW)	12.80 ± 0.22
Total sugars	% (FW)	25.61 ± 0.03
Proteins	% (FW)	0.24 ± 0.03
Ash	% (FW)	2.21 ± 0.04
Sodium (Na)	mg/100 mL	295.00
Potassium (K)	mg/100 mL	280.00
Calcium (Ca)	mg/100 mL	310.00
Magnesium (Mg)	mg/100 mL	55.00
Iron (Fe)	mg/100 mL	2.10
Zinc (Zn)	mg/100 mL	0.28
Copper (Cu)	mg/100 mL	0.05
Manganese (Mn)	mg/100 mL	0.07
Total phenolic content	mg GAE/100 g (DW)	572.0 ± 3.41

Values are expressed as mean ± standard deviation (*n* = 3). FW: fresh weight; DW: dry weight. * Titratable acidity expressed as g citric acid equivalents per 100 g FW. Total phenolic content determined by the Folin–Ciocalteu method and expressed as gallic acid equivalents (GAE).

**Table 2:** Central Composite Design (CCD): experimental runs and measured responses during acetic fermentation. Values are mean ± SD (*n* = 3).

Run	Factors		Responses							
	ADG	YE	Acetic Acid Content (%; *w*/*v*) ± SD				Residual Alcohol (%; *v*/*v*) ± SD			
			4j	8j	12j	16j	4j	8j	12j	16j
1	7.00	0.20	1.66 ± 0.03	6.48 ± 0.02	6.48 ± 0.21	6.48 ± 0.02	5.0 ± 0.00	0.25 ± 0.02	0.00	0.00
2	7.00	0.20	1.68 ± 0.01	6.50 ± 0.01	6.50 ± 0.00	6.50 ± 0.01	5.0 ± 0.02	0.25 ± 0.02	0.00	0.00
3	5.00	0.30	2.50 ± 0.01	4.00 ± 0.01	4.20 ± 0.02	4.2 ± 0.062	2.0 ± 0.00	0.5 ± 0.01	0.00	0.00
4	7.00	0.05	1.08 ± 0.00	5.00 ± 0.10	6.00 ± 0.04	6.00 ± 0.03	5.7 ± 0.01	1.00 ± 0.03	0.25 ± 0.06	0.00
5	7.00	0.20	1.66 ± 0.07	6.00 ± 0.05	6.00 ± 0.08	6.25 ± 0.02	5.0 ± 0.05	0.25 ± 0.06	0.00	0.00
6	7.00	0.20	1.68 ± 0.04	6.25 ± 0.03	6.25 ± 0.06	6.25 ± 0.07	5.4 ± 0.02	0.20 ± 0.04	0.20 ± 0.04	0.00
7	9.00	0.10	0.36 ± 0.02	1.86 ± 0.01	3.90 ± 0.16	5.03 ± 0.01	7.0 ± 0.01	4.20 ± 0.01	1.00 ± 0.02	0.5 ± 0.02
8	7.00	0.20	1.68 ± 0.06	5.94 ± 0.15	6.00 ± 0.09	6.00 ± 0.02	5.4 ± 0.02	0.20 ± 0.01	0.00	0.00
9	5.00	0.10	2.00 ± 0.02	4.00 ± 0.02	4.15 ± 0.03	4.80 ± 0.01	2.5± 0.05	0.50 ± 0.00	0.25 ± 0.06	0.00
10	4.17	0.20	2.30 ± 0.00	3.00 ± 0.01	3.5± 0.04	4.00 ± 0.02	1.0 ± 0.03	0.53 ±0.01	0.20 ± 0.01	0.00
11	9.82	0.20	0.12 ± 0.02	0.90 ± 0.08	2.00 ± 0.06	4.00 ± 0.03	7.0 ± 0.02	5.50 ± 0.01	1.50 ± 0.01	0.5 ± 0.01
12	9.00	0.30	0.40 ± 0.01	2.40 ± 0.05	2.90 ± 0.03	4.74 ± 0.026	7.0 ± 0.01	3.50 ±0.01	1.00 ± 0.03	0.00
13	7.00	0.34	1.66 ± 0.05	5.00 ± 0.20	5.0 ± 0.02	5.00 ± 0.033	5.0 ± 0.02	0.90 ± 0.01	0.5 ± 0.088	0.00

All experimental results were highly significant (*p* < 0.0001). The center point (X_1_ = 7.00% *v*/*v*; X_2_ = 0.20 g/L) was replicated five times (runs 1, 2, 5, 6, and 8), allowing an accurate estimation of pure error and improving the reliability of the regression model. ADG = alcoholic degree initial (%; *v*/*v*); YE = Yeast extract (g/L)

**Table 3:** Second-order polynomial equations describing the effect of initial alcoholic degree and yeast extract concentration on acetic acid content at different fermentation times.

Fermentation Time	Polynomial Equation
4 days	Y = 0.180 + 0.475X_1_ + 11.52X_2_ − 0.0562X_1_^2^ − 14.47X_2_^2^ − 0.575X_1_X_2_
8 days	Y = −20.39 + 7.301X_1_ + 24.73X_2_ − 0.5611X_1_^2^ − 72.00X_2_^2^ + 0.675X_1_X_2_
12 days	Y = −17.67 + 6.446X_1_ + 24.53X_2_ − 0.4581X_1_^2^ − 45.70X_2_^2^ − 1.313X_1_X_2_
16 days	Y = −8.81 + 4.039X_1_ + 10.90X_2_ − 0.2906X_1_^2^ − 41.24X_2_^2^ + 0.387X_1_X_2_

Y = acetic acid content (% *w*/*v*), X_1_ = coded initial alcoholic degree (% *v*/*v*), X_2_ = coded yeast extract concentration (g L^−1^). The negative quadratic coefficients observed for both factors indicate the existence of a maximum response, confirming the suitability of the response surface methodology for identifying optimal fermentation conditions. X_1_ and X_2_ represent coded variables corresponding to initial alcoholic degree and yeast extract concentration, respectively.

**Table 4:** Experimental design and calculated responses for fermentation efficiency (EF, %) and acetic acid productivity (AAP, g L^−1^ day^−1^) during acetic fermentation under different operating conditions.

Order	Alcoholic Degree (*v*/*v*; %)	Yast Extract (g/L)	Factors							
			EF (%)				AAP (g/L/Day)			
			4 d	8 d	12 d	16 d	4 d	8 d	12 d	16 d
1	7.00	0.200	56.02 ± 1.410	71.40 ± 0.538	68.85 ± 2.820	68.85 ± 0.269	3.65 ± 0.09	7.85 ± 0.031	5.23 ± 0.214	3.93 ± 0.015
2	7.00	0.200	56.80 ± 1.166	71.63 ± 0.399	69.07 ± 0.000	69.07 ± 0.134	3.7 ± 0.031	7.88 ± 0.015	5.25 ± 0.000	3.94 ± 0.008
3	5.00	0.300	58.84 ± 0.313	64.81 ± 0.385	61.40 ± 0.376	61.40 ± 1.166	5.75 ± 0.031	4.75 ± 0.015	3.33 ± 0.020	2.50 ± 0.047
4	7.00	0.058	51.95 ± 0.489	61.40 ± 1.943	65.95 ± 1.275	63.59 ± 0.403	2.2 ± 0.000	6.00 ± 0.153	4.83 ± 0.041	3.63 ± 0.023
5	7.00	0.200	56.13 ± 5.011	65.95 ± 1.414	63.59 ± 1.074	66.33 ± 0.269	3.65 ± 0.214	7.25 ± 0.077	4.83 ± 0.082	3.78 ± 0.015
6	7.00	0.200	71.03 ± 3.438	68.29 ± 0.907	68.29 ± 1.321	66.33 ± 0.940	3.7 ± 0.122	7.56 ± 0.046	5.04 ± 0.061	3.78 ± 0.054
7	9.00	0.100	6.14 ± 0.978	26.54 ± 0.264	35.50 ± 1.989	43.61 ± 0.236	0.4 ± 0.061	2.08 ± 0.015	3.08 ± 0.163	3.02 ± 0.008
8	7.00	0.200	71.04 ± 4.613	64.79 ± 2.190	63.59 ± 1.208	63.59 ± 0.269	3.7 ± 0.184	7.18 ± 0.230	4.83 ± 0.092	3.63 ± 0.015
9	5.00	0.100	55.29 ± 2.107	64.81 ± 0.418	63.84 ± 1.581	70.61 ± 0.188	4.5 ± 0.061	4.75 ± 0.031	3.29 ± 0.031	2.88 ± 0.008
10	4.17	0.200	50.85 ± 0.589	59.04 ± 0.457	63.80 ± 1.144	69.94 ± 0.451	5.25 ± 0.000	3.50 ± 0.015	2.75 ± 0.041	2.38 ± 0.015
11	9.82	0.200	0.00 ± 0.000	12.44 ± 1.776	16.60 ± 0.702	31.29 ± 0.344	0 ± 0.000	0.88 ± 0.122	1.50 ± 0.061	2.38 ± 0.023
12	9.00	0.300	7.68 ± 0.517	30.70 ± 0.923	25.90 ± 0.471	38.71 ± 0.272	0.5 ± 0.031	2.75 ± 0.077	2.25 ± 0.031	2.84 ± 0.020
13	7.00	0.341	56.05 ± 3.037	60.39 ± 3.203	56.69 ± 1.229	52.63 ± 0.443	3.65 ± 0.153	6.00 ± 0.306	4.00 ± 0.020	3.00 ± 0.025

**Table 5:** Comparison of functional properties of date juice and optimized date vinegar.

Sample	Total Phenolics (mg GAE/100 mL)	Antioxidant Activity (DPPH, % RSA)
Date juice	57.5 ± 0.34 ^a^	69.4 ± 1.47 ^a^
Date vinegar	62.0 ± 0.09 ^b^	78.0 ± 1.28 ^b^

For comparison purposes, phenolic contents were expressed per 100 mL of liquid matrix. Different lowercase letters (a, b) in the same column indicate significant differences (*p* < 0.05).

## Data Availability

The original contributions presented in the study are included in the article; further inquiries can be directed to the corresponding authors.
